# 

**Published:** 1918-09-28

**Authors:** 


### The Hospital

The Workers' Newspaper of Administrative Medicine and Institutional Life, Administration, National Insurance and Health.

### CONTENTS.

The Voluntary Hospitals and the War ... ... 547

A Ministry of Health for Scotland ... ... ... 548

Hospital and Institutional News ... ... ... 549
The Sherburn Hospital Foundation—Hospital Needed for Malta—The Patteran in France—A Secretary's Recollections—Shell-shock Cases and Unemployment—A Fine Cottage Hospital Chairman—Two Mayors to the Rescue—Tramway Stages and Out-patients—Medical Men on Public Bodies—Privates David and Jonathan—The Issue of Certificates in Poor-Law Infirmaries—Lambeth Guardians Explain—School Doctors and Picture Palaces—The Opposition to Welfare Centres—The Want of Dressings in Holland—An Opportunity for the Rich Druggists—Another German Ruse Frustrated—The Spanish Epidemic in Holland—This Week's Drug Market—To Our Readers.

Papers on Madness ... ... ... ... ... 553

VI. Unsound Conduct and Unsound Mind.

Relaxations and Hobbies ... ... ... ... 555

The Commonplace Book.

The Nursery School ... ... ... ... 556

The Design of the Quadrangle.

"Building New Jerusalem" ... .. ... ... 557

A Great Ideal to be Attained.

A Question of Attitude ... ... ... ... 558
The Right Policy. Choose Honest Workers and Trust Them.

The Matrons' and Sisters' Department ... ... 559
The Local Honorary Secretary.

Round the Hospitals ... ... ... ... ... 559
The Queen of Organisers—Out-patient Damage to the Children—Brighton Guardians Back Their Nurses—Servants of the King—Some Incapables and Non-patriots—15 Workers Feed 1,000 Daily.

Some London Hospital Correspondents ... ... 562
The House-Governor Makes a Defence.
A Member of the Hospital Nursing Staff Articulates.
A "London" Certificated Nurse on "London" Nurses and Their Treatment.

The Editor's Letter-Box ... ... ... ... 564
Drunk or Dying? A Common Conclusion on Insufficient Evidence.
The Guest Hospital, Dudley.
A M.O.H. on Boots or No Boots.

Matrons' and Sisters' Appointments ... ... ... vi

Passing Events and Topics ... ... ... vi

Institutional Worker ... ... ... ... ... I
Voluntary Workers and the Maternity Act.
The Milk Bill in a Small Fever Hospital.

